# The New Era of Marketing in Plastic Surgery: A Systematic Review and Algorithm of Social Media and Digital Marketing

**DOI:** 10.1093/asjof/ojad024

**Published:** 2023-02-28

**Authors:** Orr Shauly, Troy Marxen, Pedram Goel, Daniel J Gould

## Abstract

Social media has been demonstrated to serve as a critical tool for plastic surgeons, facilitating patient engagement, peer-to-peer education and learning, and outreach to the broader public community. This study aims to perform a meta-analysis of data to determine the most valuable and useful social media platforms for practicing plastic surgeons developing their practice by assessing the perceived value to the practice and quantifying return on investment. A systematic review was performed using PubMed (National Institutes of Health, Bethesda, MD). The initial search yielded 3592 articles. Sixteen articles met inclusion and exclusion criteria. It was found that patients are more likely to engage with aesthetic content rather than scientific content. Younger generations are more likely to utilize Instagram (Meta, Menlo Park, CA), Snapchat (Santa Monica, CA), and TikTok (Culver City, CA), while older generations may be more likely to utilize Facebook (Meta, Menlo Park, CA) and YouTube (San Bruno, CA). Age-specific recommendations include utilizing Instagram, Snapchat, and TikTok with emphasis on breast augmentation for patients aged 17 and 35 given this is the most common procedure performed for this age group. Patients between the ages of 36 and 70 are most likely to be engaged on Facebook, Instagram, and Facebook with liposuction being the most common procedure in this age group. For ages 70+, patients are most likely to utilize Facebook with the most common procedure performed as blepharoplasty. Effective social media marketing for the plastic surgeon considers delivering the right content and choosing the right platform. The right content and platform are critically dependent on the specific age of the audience.

**Level of Evidence: 3:**

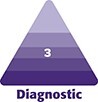

Social media is defined as the digital channel that facilitates the creation and sharing of information through virtual networks and communities.^1,2^ Over the last 2-3 decades since the dot-com bubble, social media has risen in popularity and is now ubiquitous in our lives. In its early stages, it served as a means of communicating and making connections to those far away; however, its function has since evolved, now serving as a valuable tool for corporations and businesses, providing a free and user-friendly means of outreach, advertising, and development.

According to Statista (New York, NY), a platform that provides statistics on market and consumer data, the average daily social media usage of internet users worldwide has steadily increased from 90 min per day in 2012 to 147 min per day in 2022.^3^ The inordinate amount of time spent on these platforms has paved the way for companies to adapt their business models to harness the interconnective nature of these networks.^4^ Many industries now benefit from the use of social media, including healthcare businesses, specifically surgery where a service-based practice model can exist.^4–7^

This could not be more true than for the field of plastic surgery, a crossroads where surgeons can utilize social media as an avenue for direct-to-consumer marketing, and clients/patients are seeking out their surgeons through online resources.^4^ More specifically, social media has been demonstrated to serve as a critical tool for plastic surgeons, facilitating patient engagement, peer-to-peer education and learning, and outreach to the broader public community.^4,8–15^ As such, we have seen a large shift in social media usage in plastic surgery across the last decade. In 2010, only 30% of plastic surgeons reported using social media as a source of advertising, whereas 92% of plastic surgeons reported using their practice website as a source of advertising. In addition, 62% of plastic surgeons believed social media could benefit their practice.^10^

In today's landscape, it is common to find social media pages and accounts for a plastic surgery practice. The rise in usage among plastic surgeons parallels the general rise in social media among all generations. But not all social media platforms are made equal, and their use varies by generation. Instagram (Meta, Menlo Park, CA) has developed into an increasingly popular platform for users under 35 years old, although Facebook (Meta, Menlo Park, CA) continues to be the most used platform across all age groups.^16–19^ The millennial generation (those born between 1981 and 1996) is much more likely to use Snapchat (Santa Monica, CA; temporary posts lasting a set number of seconds) and Instagram than the baby-boomer population (those born between 1946 and 1964), which is much more likely to use Facebook.^20^ These trends are seen globally in regard to plastic surgery as one study found that the United States had the most Instagram posts related to #PlasticSurgery, with over 2 million posts, 369 million likes, and 6 billion views over a 21-month period, whereas Istanbul, Turkey, was the city with the most posts (102,108).^21^ Yet despite the rise in social media engagement, only 15% of plastic surgeons post content on social media daily, whereas over 70% of millennials engage on social media numerous times each day.^22^ This discrepancy represents an untapped area that could benefit patients and plastic surgeons on numerous levels.

There is extensive literature describing the utility of social media to a plastic surgery practice; however, to our knowledge, no study has compared each social media channel to determine the platform with the greatest value to a plastic surgeon's practice. Herein, the aim of this study was to perform a systematic review to determine the most valuable and useful social media platforms for practicing plastic surgeons developing their practice by assessing the perceived value to the practice and proposing an algorithm for useful marketing strategies.

## METHODS

**Figure 1.**
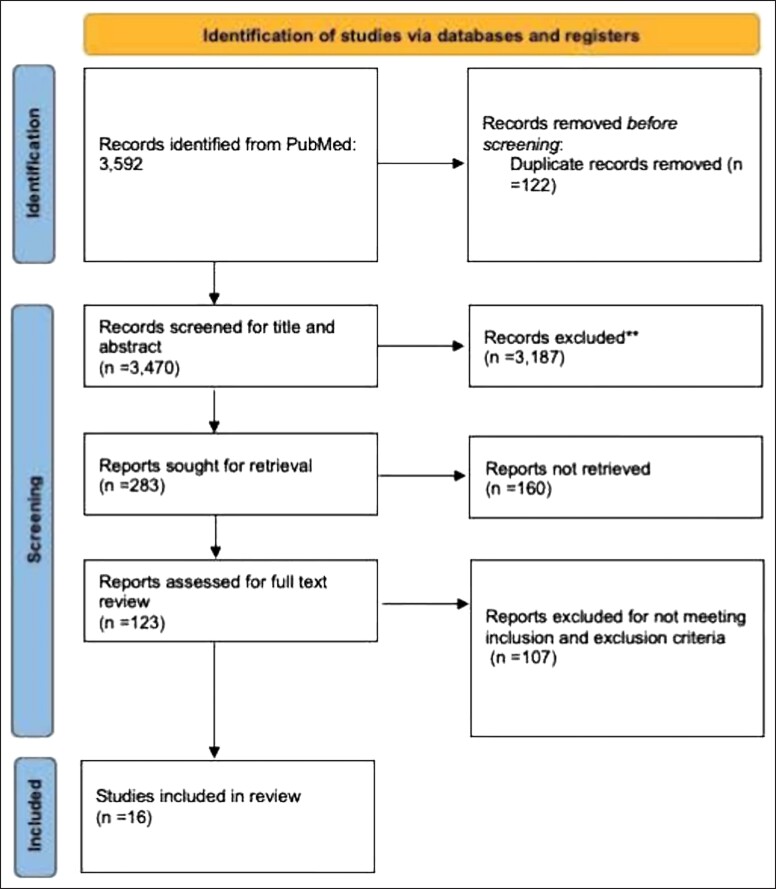
Systematic review process.

To better understand the impact of social media marketing on plastic surgery, a systematic review was performed. The review was performed by 2 authors, T.M. and O.S., over the electronic database PubMed (National Institutes of Health, Bethesda, MD) between August 1, 2022 and August 7, 2022. The initial search yielded 3592 articles. After exclusion of duplicate articles, there were 3470 articles matching our initial criteria. After reviewing the title and abstracts, there were 283 articles, and 123 articles were selected for full-text review. Disagreements were handled by review of the manuscript by the senior author (DJG). In total, 16 articles met the inclusion and exclusion criteria. Inclusion criteria consisted of studies evaluating the efficacy of social media marketing on patient engagement in plastic surgery. Exclusion criteria consisted of studies that did not quantify social media engagement or evaluated other sources of marketing on patient engagement—television, radio, and word of mouth (Figure 1).

## RESULTS

A total of 16 studies were included in our systematic review, which were organized into 2 categories. Six studies were included in content recommendations,^19,23–27^ and 12 studies were included in platform recommendations.^8,19,27–36^ Two studies were included in both categories.^19,27^ Of the studies included under content recommendations, the positive recommendations included using descriptive keywords such as “aesthetic,” “cosmetic,” and “reconstruction.” The studies also recommended focusing on aesthetic or disease components without highlighting negative images of disfiguring illnesses. It was recommended to wear a white coat and a smile in social media content. Delivering content through live video may be optimal when utilizing Facebook as the preferred platform. Factors such as humor, celebrities, and attractive female plastic surgeons may increase engagement. It is also preferable to have the surgeon as the primary messenger (Table 1).

**Table 1.** Social Media Marketing Content Recommendations for the Plastic Surgeon

Author	Year	Study	Platform	Recommendations/findings
Almarghoub et al^23^	2020	Plastic Surgery on YouTube	YouTube (San Bruno, CA)	Use phrases such as “Plastic surgery,” “breast augmentation,” and “nose jobs”
Klietz et al^24^	2020	Social Media Marketing: What Do Prospective Patients Want to See?	Instagram (Menlo Park, CA)	Emphasize aesthetic or disease components. Avoid scientific content.
Park et al^25^	2020	Building Your Brand: Analysis of Successful Oculoplastic Surgeons on Social Media	Instagram	Wear white coat and smile in social media content. Avoid office procedures.
Chopan et al^26^	2019	Plastic Surgery and Social Media: Examining Perceptions	Twitter (San Francisco, CA)	Use words such as “cosmetic,” “aesthetic,” and “reconstruction.” Avoid “plastic.”
Nayyar et al^19^	2019	Are You on the Right Platform? A Conjoint Analysis of Social Media Preferences in Aesthetic Surgery Patients	Facebook (Menlo Park, CA), Instagram, Twitter, etc.	Surgeons delivering information through live video is optimal.
Naftali et al^27^	2018	Plastic Surgery Faces the Web: Analysis of the Popular Social Media for Plastic Surgeons	Facebook, Instagram, YouTube	Celebrities, humor, attractive female plastic surgeons generate higher engagement.

Of the studies included under platform recommendations, the most evaluated platforms included Facebook, Instagram, YouTube (San Bruno, CA), Snapchat, TikTok (Culver City, CA), and Twitter (San Francisco, CA). Instagram was found in a majority of studies to generate the highest engagement, especially among younger audiences.^8,19,27,28,30,33–36^ YouTube and TikTok were found to have high engagement, but overall low-quality content.^28,29,31,32^ One study that utilized crowd-sourcing found Facebook to be the optimal platform for engagement.^19^ Another study found TikTok to generate the highest engagement^28^ (Table 2).

**Table 2.** Social Media Marketing Platform Recommendations for the Plastic Surgeon

Author	Year	Study	Platform	Recommendations/findings
Ravikumar et al^28^	2021	Is TikTok the New Instagram? Analysis of Plastic Surgeons on Social Media	TikTok (Culver City, CA), Instagram (Menlo Park, CA)	TikTok generates higher engagement than Instagram, and has the potential to engage a younger generation.
Om et al^29^	2021	Analyzing the Quality of Aesthetic Surgery Procedure Videos on TikTok	TikTok	TikTok generates high engagement but contains low-quality content.
Skrzypczak et al^30^	2021	Association between the Desire for Breast Augmentation and Instagram Engagement: A Cross-Sectional Survey among Young Polish Women	Snapchat (Santa Monica, CA), Instagram	Instagram and snapchat may predict a desire to undergo cosmetic procedures.
Ward et al^31^	2020	YouTube for Cosmetic Plastic Surgery: An Effective Patient Resource?	YouTube (San Bruno, CA)	YouTube generates high engagement but contains low-quality content.
Gray et al^32^	2020	Can You Trust What You Watch? An Assessment of the Quality of Information in Aesthetic Surgery Videos on YouTube	YouTube	YouTube videos regarding common plastic surgery procedures are of low-quality content.
Zahedi et al^33^	2020	Social Media's Influence on Breast Augmentation	Facebook (Menlo Park, CA), Instagram	Instagram may influence a patient's desire to undergo breast augmentation.
Nayyar et al^19^	2019	Are You on the Right Platform? A Conjoint Analysis of Social Media Preferences in Aesthetic Surgery Patients	Facebook, Instagram, Twitter, YouTube	Facebook is the best platform for the plastic surgeon.
Alghonaim et al^34^	2019	Social Media Impact on Aesthetic Procedures Among Females in Riyadh, Saudi Arabia	Instagram, Snapchat, Twitter	Instagram, followed by snapchat, were found to be the most influential platforms.
Naftali et al^27^	2018	Plastic Surgery Faces the Web: Analysis of the Popular Social Media for Plastic Surgeons	Facebook, Instagram, YouTube	YouTube is ideal for educational content. Instagram is ideal for self-promotional content.
Mullens et al^35^	2018	#PlasticSurgery: A Comparative Deep Dive Analysis into Social Media and Plastic Surgery	Instagram, Twitter	Instagram generates higher engagement than Twitter.
Gould and Nazarian^8^	2018	Social Media Return on Investment: How Much is it Worth to My Practice?	Instagram	Instagram is preferable to alternative platforms for generating revenue for your practice.
Sorice et al^36^	2017	Social Media and the Plastic Surgery Patient	Facebook, Instagram, Snapchat, Twitter, YouTube, etc.	Facebook has the greatest patient use and engagement. Instagram is second in terms of engaged users. Twitter has the least engagement.

### Age-Specific Social Media Marketing Guidelines

**Figure 2.**
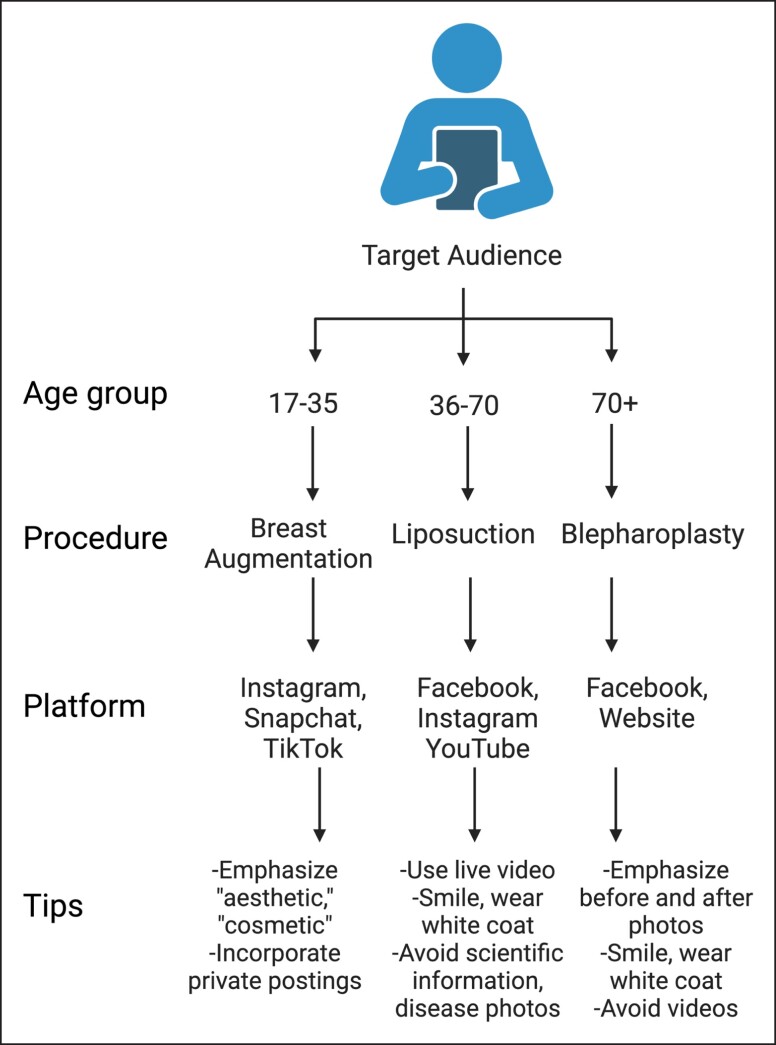
Age-specific social media marketing algorithm for a plastic surgery practice.

When incorporating both content and platform recommendations into the most recently available Aesthetic Society procedural statistics,^37^ a marketing guidance picture starts to come into form. This picture encompasses age-related content and platform recommendations (Figure 2).

According to The Aesthetic Society, between the ages 17 and 35, the most common procedure performed is breast augmentation.^37^ Given that this generation is more likely to be found on Instagram, Snapchat, and TikTok, an ideal social media marketing strategy for this age group may include utilizing these platforms to emphasize the aesthetic components of breast augmentation. It may also be prudent to advertise private postings, as this content may be perceived as likable and relatable to the younger generations. Between the ages 36 and 70, the most common procedure performed is liposuction.^37^ This generation is more likely to be engaged on Facebook, Instagram, and YouTube. To effectively market to this generation, it may be important to smile, wear a white coat, and avoid overly scientific information, focusing on the safety and education of liposuction.

Lastly, for ages 70+, the most common procedure performed is blepharoplasty.^37^ These patients may be less likely to use Instagram and TikTok but may be engaged on Facebook. The 70+ age group may also prefer before and after photographs over procedural videos of blepharoplasty. It is important in this generation to smile and wear a white coat, as this behavior is deemed professional and may be crucially important in establishing rapport and credibility.

### Social Media Marketing Content Recommendations

There was significant variety in findings across these studies; however, a consistent theme emerged of focusing on aesthetic attributes, displaying professionalism, and making sure the surgeon was the person delivering the content. Chopan et al found that among Twitter searches, certain words were associated with higher positivity scores. The words associated with higher scores were “cosmetic,” “aesthetic,” and “reconstruction.” In contrast, the word “plastic” was associated with more negative scores.^26^ Almarghoub et al found that among the same number of plastic surgery videos on YouTube, searches for “plastic surgery,” followed by “breast augmentation” and “nose jobs” yielded the highest number of views.^23^ Although searches for the keyword “plastic surgery” yielded the most results, using the word “plastic” alone was associated with a more negative score, and as such should be isolated to metadata for search purposes only. Klietz et al discovered that patients were much more likely to engage plastic surgery content with aesthetic more than scientific content. This study also emphasized the importance of private postings from plastic surgeons to give insight into their daily life.

Aesthetic posts were most likely to be saved while private posts were most likely to attract clicks and followers.^24^ Nayyar et al found that the ideal method of social media for the aesthetic patient was through live video on Facebook from a plastic surgeon.^19^ Although it should be noted that the lines distinguishing Facebook and Instagram are becoming increasingly blurred, as Instagram is now owned by Facebook, and content can be simultaneously posted on both platforms. The type of content preferred by the aesthetic plastic surgery patient may also depend on the location of the body where the procedures are performed. In sensitive procedural areas, including the eyes, patients may prefer photograph content over video content.^25^

### Social Media Marketing Platform Recommendations

Similarly to preferred content, the preferred platform for social media marketing varied and may be dependent on age. Younger generations may be more likely to utilize Instagram, Snapchat, and TikTok, whereas older generations may be more likely to utilize platforms such as Facebook and YouTube.^16–20^ Naftali et al analyzed 3 social media platforms and found that Instagram had the highest percentage of plastic surgery posts from plastic surgeons and that most of the content provided on Instagram was self-promotional, in nature. In this study, it was also discovered that posts from a celebrity were most likely to generate likes, comments, shares, and views. The posts most likely to generate attention were those posted on Instagram with personal stories, education, videos, and celebrity involvement, if possible.^27^ Mullens et al compared Twitter and Instagram for average engagement on each post related to plastic surgery, finding significantly higher traffic on Instagram as opposed to Twitter.^35^ Ward et al evaluated the quality of videos on YouTube and found that despite high engagement, the overall quality of videos, measured by DISCERN score (an instrument for judging the quality of written consumer health information on treatment choices), showed low quality and high bias.^31^ Gray et al found similar when evaluating videos of the 12 most common aesthetic surgical procedures on YouTube, the videos were of low quality.^32^ Gould and Nazarian found that Instagram and direct-to-consumer marketing were preferable to alternative platforms in terms of generating return on investment (ROI) for a practice.^8^

Ravikumar et al found that when searching among common plastic surgery hashtags, TikTok had a greater mean total engagement per post, with an emphasis on humorous and educational content, as opposed to self-promotion or personal content. TikTok, as a social media platform on the rise, has the potential to engage the next generation of individuals interested in learning more about plastic surgery.^28^ Om et al found that despite high engagement among TikTok users in general for plastic surgery content aesthetic procedures, the average quality of videos, as measured by DISCERN scores, was relatively low.^29^ Nayyar et al found that the ideal method of social media for the aesthetic patient was through live video on Facebook from a plastic surgeon.^19^ In a survey of plastic surgery patients, Facebook had the greatest patient use and engagement. YouTube came in second place. Instagram was second in terms of engaged users. Twitter was the least popular network, with the least number of users and the lowest levels of engagement. Articles from the plastic surgeon generated the least engagement while before and after photographs and practice information were much more likely to generate interest.^36^

Snapchat use and Instagram engagement may predict a desire for cosmetic procedures.^30^ Instagram is the most powerful tool that influences patients’ desire to undergo breast augmentation.^33^ Alghonaim et al found that 81% of patients that visited a facial plastics clinic were between the ages 25 and 34. 97% found that cosmetic social media accounts were helpful. 78% found that the information on the accounts was not sufficient. 68% reported that the accounts had an influence on them. Instagram, followed by Snapchat, were found to be the most influential platforms.^34^

## DISCUSSION

Through social media, plastic surgeons can not only advertise more easily, but they can also educate, disseminate research, and engage with patients more effectively.^38^ Patients, on the other hand, can more easily identify a plastic surgeon that meets their needs, more readily inquire and ask questions, and get a feel for their bedside manner before walking in the doors of their office.

Despite these potential benefits, there has been a reluctance among some plastic surgeons to endorse social media. Social media use has been more common among private practitioners, as it may be more geared toward patient acquisition and branding.^39^ Plastic surgeons with an aesthetic-focused practice are more likely to use social media and most believe it is an effective marketing tool, especially due to the visual components embedded in most social media platforms.^22^ Among plastic surgeon nonusers, they may feel that social media requires too much time and resources, or may risk breakdowns in patient privacy.^40^ Although academic plastic surgeons have been more reticent to engage on social media, there has been an exponential rise in Instagram usage by plastic surgery residency programs since 2015.^41–43^ Increasing a program's social media presence has been shown to increase case and procedure volume in plastic surgery clinics, including resident clinics.^44^ Whether academic programs embrace social media or not, there are many benefits to using social media properly, and patients are consistently turning to social media for information on plastic surgery. Nearly two-thirds of people in a survey believed that social media before and after pictures have an influence on the trend of cosmetic procedures performed.^45^ Roughly half of the patients undergoing cosmetic procedures report being influenced by social media to consider undergoing cosmetic procedures and around the same percentage reports following plastic surgeons on social media.^46^

In patients who will eventually undergo an aesthetic plastic surgery procedure, nearly all use the internet before their initial visit. Google front page placement for plastic surgery has been shown to be related to social media following on platforms such as Facebook, Twitter, and Instagram. It is not, however surprisingly, not related to medical school ranking or years in practice.^47^ Google is also the first place people would go to find a plastic surgeon, follow by the surgeon's practice website or social media platforms as the next most influential aspects in selecting a plastic surgeon.^18^ Social media strongly influences up to 40% of these patients when deciding what plastic surgeon to select, despite the wide range of information quality available online and throughout social media platforms.^48^ In fact, upon analysis of millions of plastic surgery-related posts, only 4% of these posts can be linked to board-certified plastic surgeons eligible for membership in The Aesthetic Society.^49^ This stunning reality illustrates the need for more private and academic board-certified plastic surgeons to embrace social media.

Ultimately, the limitations of this review reflect those of the currently available literature, with limited high-quality data currently published. Even so, social media continues to be a force in influencing the general public's perception of plastic surgery.^19,50^ It is up to the plastic surgeon to be mindful of how they choose to market their services to the public. Increasingly, former methods of marketing, such as television, radio, and word-of-mouth, are being replaced by popular social media platforms such as Instagram, which can have powerful effects on the revenue of one's practice.^8^ Surgeons may use the data herein to guide their marketing strategy. This manuscript provides a framework for targeted marketing to those demographics most aligned with a surgeon's practice. In addition, resource allocation may be adjusted accordingly to target those platforms that are recommended herein.

## CONCLUSIONS

While social media use and following is not a well-established predictor for the ability to disseminate research, it remains a powerful source of marketing in today's landscape. Effective social media marketing for the plastic surgeon considers delivering the right content and choosing the right platform. Additionally, the right content and platform are critically dependent on the specific age of the audience. When taken together, social media marketing can be a powerful tool to engage and educate the public, while simultaneously increasing the revenue of one's practice. We believe all plastic surgeons can and should be open to incorporating various methods of social media into their marketing strategies.

## Data Availability

Data is available for review on request.
